# Assessing Socioeconomic Vulnerabilities Related to COVID-19 Risk in India: A State-Level Analysis

**DOI:** 10.1017/dmp.2020.348

**Published:** 2020-09-10

**Authors:** Praveen Kumar Pathak, Yadawendra Singh, Sandhya R. Mahapatro, Niharika Tripathi, Jyoti Jee

**Affiliations:** Department of Geography, Faculty of Natural Sciences, Jamia Millia Islamia (Central University), New Delhi, India; Department of Economics, Chandradhari Mithila College, Lalit Narayan Mithila University, Darbhanga, Bihar, India; A.N. Sinha Institute of Social Studies, Patna, Bihar, India; ICMR-National Institute of Medical Statistics, New Delhi 110029, India; Post-Graduate Department of Geography, Veer Kunwar Singh University, Ara, Bihar, India

**Keywords:** pandemics, epidemiologic methods, geographic mapping, policy making, public health

## Abstract

**Objective:**

There is a paucity of scientific analysis that has examined spatial heterogeneities in the socioeconomic vulnerabilities related to coronavirus disease 2019 (COVID-19) risk and potential mitigation strategies at the sub-national level in India. The present study examined the demographic, socioeconomic, and health system-related vulnerabilities shaping COVID-19 risk across 36 states and union territories in India.

**Methods:**

Using secondary data from the Ministry of Health and Family Welfare (MoHFW), Government of India; Census of India, 2011; National Family Health Survey, 2015-16; and various rounds of the National Sample Survey, we examined socioeconomic vulnerabilities associated with COVID-19 risk at the sub-national level in India from March 16, 2020, to May 3, 2020. Descriptive statistics, principal component analysis, and the negative binomial regression model were used to examine the predictors of COVID-19 risk in India.

**Results:**

There persist substantial heterogeneities in the COVID-19 risk across states and union territories in India. The underlying demographic, socioeconomic, and health infrastructure characteristics drive the vulnerabilities related to COVID-19 in India.

**Conclusions:**

This study emphasizes that concerted socially inclusive policy action and sustained livelihood/economic support for the most vulnerable population groups is critical to mitigate the impact of the COVID-19 pandemic in India.

The novel coronavirus disease 2019, also referred to as COVID-19, is a global health emergency that has triggered an unprecedented catastrophe with respect to human lives and livelihood, disrupted economic systems cutting across sectors, halted public transportation networks, and restricted social interactions across the globe. On March 11, 2020, the World Health Organization (WHO) announced COVID-19 as a pandemic and called for decisive action, including the need to: (i) prepare and be ready; (ii) detect, protect, and treat; (iii) reduce transmission; (iv) innovate and learn.^1^ Since late December 2019, when early clusters of COVID-19 cases were reported from Wuhan City, Hubei Province of the People’s Republic of China, more than 9.84 million confirmed COVID-19 cases of infection and 495,760 COVID-19 related deaths have been recorded across 216 countries and territories.^2^ The impact of COVID-19 has been devastating across the globe, although its repercussions may be more serious for the developing countries characterized by a relatively large population base, great strain on the available resources (energy, food, water, land), inadequate public health infrastructure, fragile economic systems, and weak social safety programs.^3^

Several national governments and global agencies have been struggling to tackle the COVID-19 pandemic,^4^ seeking to contain the burgeoning risk of infection, fatality rate, improve access to affordable health care, ensure food security, and the protection of livelihood opportunities among the most vulnerable population groups (eg, children, elderly, pregnant women, people with co-morbidities, people with disabilities, migrants, slum dwellers).^5-10^ Some of these vulnerable population groups are also the least likely to be able to practice the preventive measures related to physical distancing and self-isolation given their health and livelihood constraints.^11^ Moreover, governments in many of these countries do not have enough resources for quarantine facilities.^12-14^

It is important to note that any disaster, natural or man-made, spreads indiscriminately, although it disproportionately impacts the human population along the contours of socioeconomic and gendered inequities in the affected region.^15,16^ For instance, recent evidence has highlighted the disproportionate burden of COVID-19 risk among those aged 60 years and above; those afflicted with co-morbidities; disabilities, and poor nutritional status, as well as other marginalized groups.^17-19^ Therefore, regular assessment of underlying demographic, socioeconomic, epidemiological, and environmental exposure to COVID-19 risk is critical for an effective management and mitigation strategy, particularly among the low-and-middle-income countries.^20-22^

India is among the top 10 countries most impacted by COVID-19 and has been fighting the pandemic since the first confirmed case was reported in January 2020 in Thrissur, one of the districts of the southern state of Kerala.^23^ After the WHO declared COVID-19 a pandemic, the Union Government of India invoked the Disaster Management Act 2005 to implement a 21-day complete nationwide lockdown from March 25, 2020, to April 14, 2020 (Lockdown 1), to contain the spread of COVID-19.^24^ This was probably done to “flatten the epidemic curve” and to manage the contagion of the pandemic in such a way that it allows the public health system to meet the substantial surge of patients.^25^ Subsequently, the nationwide lockdown was extended from April 15, 2020, to May 3, 2020 (Lockdown 2); from May 4, 2020 to May 17, 2020 (Lockdown 3); and from May 18, 2020 to May 31, 2020 (Lockdown 4) with gradual relaxation of lockdown conditions in a phased manner after continuous review of the emergency situation. The gradual slackening of lockdown, particularly in the low-burden states and union territories, was provided to facilitate the revival of economic activities and routine human lives.^26^

Given the large and relatively dense population base, a rising share of aging population with a poor health-care system, regional inequalities in economic development and higher population mobility,^27-29^ India appears to be at greater risk of an increasing number of COVID-19 infected people. Identification of risk factors that are responsible for the rise/decline in the rate of disease spread is critical to provide direction for effective and long-term planning. It is opined that socioeconomic and environmental factors play a significant role in influencing the prevalence of any disaster including COVID-19.^30^ Although demographic pressures and geographical vulnerabilities enhance exposure to the disease, many pre-existing vulnerabilities, such as age structure, morbidity, and poor environmental conditions also contribute to the spike in the spread of the pandemic. On the other hand, the resilience capacity of the state and communities, such as improved health infrastructure, better working conditions, and economic status, may enable them to contain the disease spread.^31^ Indian states are positioned at different stages of socioeconomic and demographic transition; hence, the vulnerability to COVID-19 risk may vary between and within states. One can expect that the vulnerability to COVID-19 would be high in the relatively laggard states of the country given their poorer socioeconomic and health infrastructure status.^32^

The present study attempts to examine the demographic, socioeconomic, and health system-related vulnerabilities against COVID-19 risk across 36 states and union territories in India. Furthermore, a composite measure of socioeconomic vulnerabilities has been generated by incorporating a diverse set of indicators ranging from demographic and socioeconomic characteristics to others such as health infrastructure, which may allow a comprehensive understanding of risk factors that augment the vulnerability to COVID-19 pandemic at the sub-national level in India.^33^ Findings of the present study may facilitate a long-term plan of action for containing the transmission of this pandemic. To the best of our knowledge, this is the first study to investigate the sub-national level vulnerabilities shaping COVID-19 risk across states and union territories in India.

## METHODS

### Data

Data used for the study were compiled from multiple sources, such as the Ministry of Health and Family Welfare, Government of India- 2020, COVID19India.org, Census of India- 2011, National Family Health Survey- 2015-2016, National Sample Survey 68th round- 2011-12, National Sample Survey 75th round- 2017-18, and National Sample Survey 76th round- 2018, Periodic Labour Force Survey- 2017-2018, and National Health Accounts- 2018.^34-43^

### Study Design

The confirmed positive COVID-19 cases have been used as the main outcome variable in the study. The unit of analysis for the study is all 36 states and union territories of India. Table 1 provides a detailed list of demographic, socioeconomic, and health system-related indicators, data sources, and description of the indicators. We reviewed different frameworks, ranging from the infectious disease vulnerability index, socioeconomic vulnerability indicator framework, and recent COVID-19 vulnerability assessment tools (focused on socioeconomic inequalities, population characteristics, access to services, and epidemiological factors), to investigate the sub-national vulnerability assessment of COVID-19 risk in India.^33,44,45^ The infectious disease vulnerability framework identified 7 domains (demographic, health care, public health, disease dynamics, political-domestic, political-international, and economic), which show a nation’s/region’s ability to prevent or contain a disease outbreak. We adopted the modified infectious disease vulnerability framework according to its suitability and feasibility as per available data in the Indian context. The COVID-19 vulnerability assessment is performed using the selected domains related to demographic composition, socioeconomic structure, disease dynamics, health care, and public health system characteristics across 36 states and union territories in India.

**TABLE 1:** Description of the Study Variables

Risk Factors	Description of Variables	Data Source
Outcome variable	Number of COVID-19 cases	Ministry of Health and Family Welfare, Government of India; www.covid19india.org
Demographic composition	• Percent of Scheduled Caste/Scheduled Tribe population • Percent of Muslim population • Percent of elderly population (60 y and above) • Percent of literate population • Number of employment related inter-state migrants during the past 5 y • Number of employment related international migrants during the past 5 y • Percent of slum population • Percent of urban population • Population density (number of persons per square km)	Census of India, 2011
Disease dynamics	• Co-morbidity (at least 1 ailment per 1000 population). The diseases considered in the analysis are HIV AIDS, cancer, diabetes, other endocrine, metabolic, nutritional diseases including obesity, hypertension, heart disease, and bronchial asthma.	National Sample Survey, 75^th^ round, 2017-18
Health care and public health	• Percent washing hand before meal • Percent washing hand after defecation • Percent household used for purpose other than residential • Percent of households bringing water from outside household premise • Average people living per room • Percent of joint/extended family	National Sample Survey, 76^th^ round,2018
	• Health infrastructure Index constructed using average population covered per primary health center (PHC), community health center (CHC), sub-centre, district hospital, auxiliary nursing midwife (ANM), doctors, hospital-bed ratio • Per capita health expenditure	National Health Profile, 2018 National Health Profile, 2015
Socio-economic structure	• Mean score of mass media exposure based on the relative frequency of watching television/listening radio/reading newspaper	National Family Heath Survey, 2015-16
	• Percent living below the poverty line	National Sample Survey, 68^th^ round, 2011-12
	• Percent casual laborers in agriculture • Percent casual laborers in non-agriculture	Periodic Labour Force survey, 2018

### Methods

We have applied bivariate and multivariate techniques to understand the sub-national level vulnerabilities associated with COVID-19 risk in India. The main outcome variable of the study is the number of COVID-19 positive cases for the period March 16, 2020, to May 3, 2020. We restricted the analysis to this date range because the national lockdown was implemented across the country with a comprehensive restriction on population movement between states and union territories. For the descriptive analysis, we classified the states and union territories into 3 broad categories (high-, medium-, and low-burden states) based on the number of COVID-19 positive cases seen in each case. For instance, states and union territories with more than 3000 positive cases until May 3, 2020, were categorized into a high-burden cluster, those with 1000 to 3000 COVID-19 positive cases were categorized as a medium-burden cluster, and others with less than 1000 COVID-19 positive cases were categorized as a low-burden cluster (Appendix 1).

We used COVID-19 positive cases, prevalence rate, positivity rate, recovery rate, and case-fatality rate to monitor the sub-national trends related to the COVID-19 pandemic in India during the study period. The exponential growth method was used to project the state and union territories total population using data from the Census of India, 2011, to estimate the COVID-19 prevalence rate and recovery rate per million population.^46^ A complete description of variables, measurements, and data sources is presented in Table 1. We used principal component analysis to compute the “health infrastructure index” based on an array of health systems performance variables such as average population served per hospital, community health center, primary health center, sub-center, doctors, auxiliary nursing midwife, and hospital bed ratio.^47,48^ The negative value of the index suggests “high vulnerability,” whereas the positive value of the index indicates “low vulnerability.” The reliability of the index was assessed using Cronbach’s alpha test.^49^

We used a negative binomial regression model to identify the association of various demographic, socioeconomic, and health system-related factors with the COVID-19 pandemic. A Poisson regression model is a commonly used model for count data. The assumption of the Poisson model is that variance is equal to mean. However, due to unobserved heterogeneity and clustering, data often present an over dispersion. The negative Binomial regression model, however, relaxes the equi-dispersion restriction of the Poisson model.^50^ Negative Binomial regression model typically uses the log link to relate the mean of the data to the set of covariates. The regression model with log odds link function is given by:

η = β_0_ + β_1_X_1_ + β_2_X_2_ + β_3_X_3_+……….+ β_k_X_k_

where “η” is log of the outcome variable that is the number of COVID-19 positive cases.

To summarize, from the sub-national heterogeneities related to the socioeconomic vulnerability to the COVID-19 pandemic in India, we generated 4 different sets of vulnerabilities indices (demographic susceptibility index, socioeconomic and disease exposure index, public health resilience index, and composite vulnerability index) using the principal component analysis. We mapped COVID-19 risk-related vulnerability indices, prevalence rate, and recovery rate across states and union territories using ArcGIS software. Line graphs were plotted to present the trends of positive cases, positivity rate, recovery rate, deaths, and case-fatality rate related to COVID-19. We performed all statistical analyses using STATA 13.0 software.

## RESULTS

### Sub-national Trends of COVID-19 in India

**FIGURE 1:**
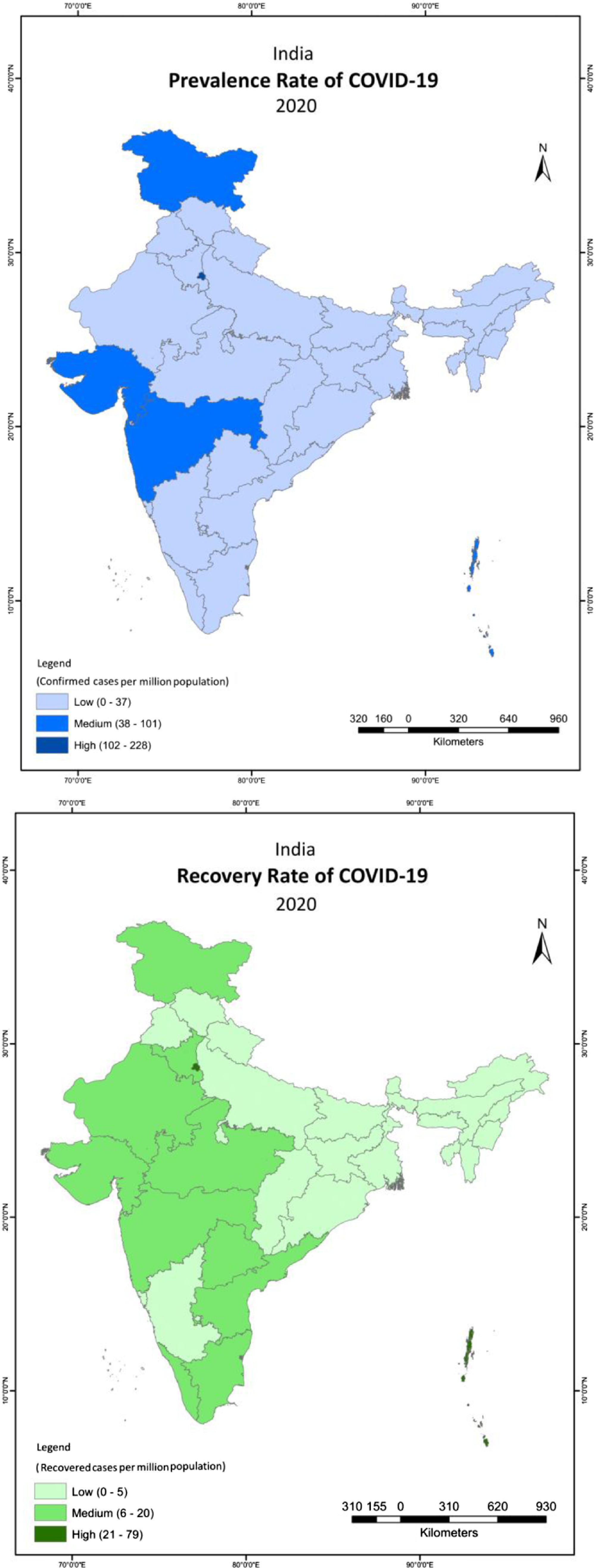
Prevalence of COVID-19 Positive Cases and Recovered Cases of COVID-19 Across States and Union Territories in India

From March 16, 2020, to May 3, 2020, the prevalence rate of COVID-19 positive cases was 30 per million population in India with substantial inter-state differentials. For instance, 10 states and union territories had higher prevalence rates of COVID-19 positive cases than the national average, which included among others, Delhi (228), Maharashtra (101), Gujarat (77), Tamil Nadu (37), Rajasthan (35), and Madhya Pradesh (33) (Figure 1). The prevalence of recovered cases of COVID-19 in India was 8 per million population, marked by substantial inter-state variability during the period of March 16, 2020 to May 3, 2020.

**FIGURE 2:**
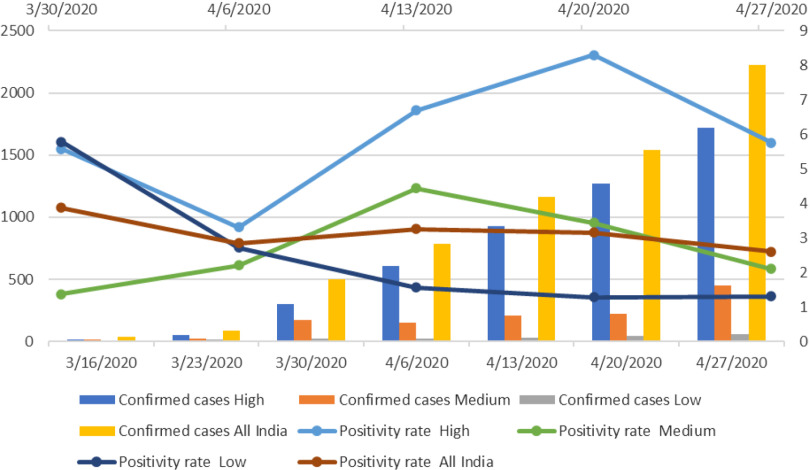
Average Weekly Confirmed Cases and Positivity Rate in Low, Medium and High COVID-19 Burden States From March 16, 2020, to May 03, 2020, India

The sub-national average weekly COVID-19 positive cases, and the corresponding positivity rate suggest that the average number of weekly new confirmed COVID-19 positive cases have increased continuously across states and union territories of India (Figure 2). On an all India basis, the cases increased exponentially from 41 on March 16, 2020, to 2227 on April 27, 2020. We calculated the positivity rate, which is the percent of confirmed COVID-19 positive cases out of the total tested case. The positivity rate has been presented from March 30, 2020 when data of COVID-19 testing became available (Figure 2). The positivity rate declined from 3.9 per cent to 2.9 per cent during the first week (March 16, 2020, to March 23, 2020) in India. After April 06, 2020, there was not much variation in the all India positivity rate; it was 2.6 per cent for the last week of our analysis (April 27, 2020, to May 3, 2020).

**FIGURE 3:**
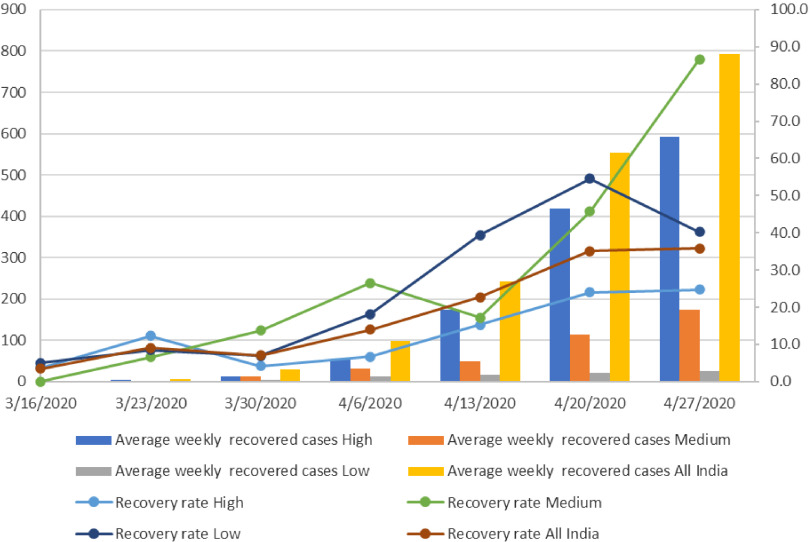
Average Weekly New Recovered Cases and Recovery Rate in Low, Medium and High COVID-19 Burden States From March 16, 2020, to May 03, 2020, India

Subsequently, the average weekly recovered cases and recovery rate from COVID-19 has been analyzed (Figure 3). It is observed that the average weekly recovered cases have increased continuously for all the categories in states across India. The all India average weekly recovered cases increased from 1 in the first week to 792 in the last week. Data suggest that recovery rate, (recovered cases as a percentage of total COVID-19 positive cases), has improved for India and all its states.

**FIGURE 4:**
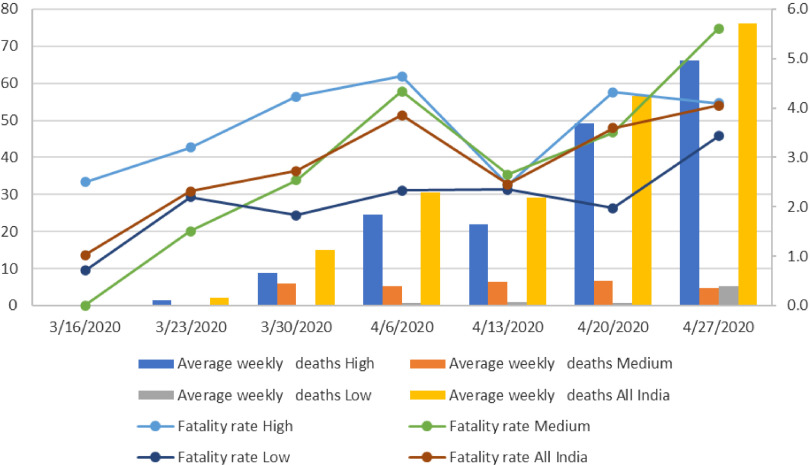
Average Weekly Deaths and Fatality Rate in Low, Medium, and High COVID-19 Burden States From March 16, 2020, to May 03, 2020, India

While examining the COVID-19 related deaths and the case-fatality rate across states, it emerged that the average weekly deaths due to COVID-19 increased continuously over time (Figure 4). The average weekly deaths increased from none during the first week (March 16, 2020) to 76 during the week of April 27, 2020, in India. It was observed that of these 76 COVID-19 deaths, 66 occurred in the high category states, while the remaining casualties occurred in the low category states.

### Sub-national Analysis of Different Dimension of the Socioeconomic Vulnerabilities Related to COVID-19 Positive Cases

We analyzed the patterning of COVID-19 positive cases across a set of selected factors such as demographic susceptibility, socioeconomic, and disease exposure, and public health resilience capacity characteristics at the sub-national level in India (Tables 2-4). The sub-national analysis of demographic susceptibility patterns related to COVID-19 shows that states such as Maharashtra, Gujarat, Delhi, and Tamil Nadu, which recorded the highest burden of COVID-19 positive cases, also happened to have relatively higher employment-related in-migration (Table 2). On the other hand, the north and eastern states, including Bihar and Jharkhand, belonging to the low COVID-19 category, reported a smaller volume of migrants. A similar pattern was noticed concerning international migration as well. The association between level of economic development and prevalence of the COVID-19 pandemic shows that states having a higher burden of infections seemed to have a low and moderate level of poverty, whereas around 43% of laggard states reported a low level of infection and had a high level of poverty.

**TABLE 2:** Sub-national Analysis of COVID-19-Related Demographic Susceptibility Patterns in India

Characteristics	Low-Burden COVID-19 Cluster	Moderate-Burden COVID-19 Cluster	High-Burden COVID-19 Cluster
**Inter-state migration**			
Low	BR, JK, AN, PY, AN, ML, MN, AR, MZ (42.9)	(0.0)	(0.0)
Middle	WB, KL, OR, JH, CH, UT, CT, AS, HP, GA (47.6)	(0.0)	(0.0)
High	KR, HR (9.5)	RJ, MP, UP, AP, PB, TG (100.0)	MH, DL, GJ, TN (100.0)
**International migration**			
Low	JK, AN, PY, NE (38.1)	(0.0)	(0.0)
Middle	BR,OR, JH,CH,CTGA,MZ(33.3)	RJ, MP, AP, TG (66.7)	(0.0)
High	WB, KR,KL,HR,UT, HP ((28.6)	UP, PB (33.3)	MH, DL, GJ, TN (100.0)
**Casual labor in non-agriculture**			
Low	CH, UT, GA, MN, AR, MZ (28.6)	(0.0)	MAH, DEL, GJ (75.0)
Middle	JK, KR, BR, HR, AS, AN, TR (33.3)	RJ, UP, PB, TG (75.0)	(0.0)
High	WB, KL, OR, JH, CT, HP, ML, PY (38.1)	MP, AP (25.0)	TN(25.0)
**Poverty**			
Low	KL, HP, AN, PY, GA (23.8)	AP, TG, PB (50.0)	DL (25.0)
Middle	WB, JK, HR, UT, TR, MG, MZ (33.3)	RJ (16.7)	TN, GJ, MH (75.0)
High	JH, BR, OR, CH, CT, AS, MN, AR (42.9)	MP, UP (33.3)	(0.0)
**Elderly population**			
Low	CH, AS, AN, MG, MN, AR, MZ (33.3)	(0.0)	DL (25.0)
Middle	WB, JK, BR, HR, JH, CT, TR (33.3)	RJ, MP, UP (50.0)	GJ (25.0)
High	KR, KL, OR, UT, HP, PY, GA (33.3)	AP, TG, PB (50.0)	MH, TN (50.0)
**Ailment**			
Low	BR, JH, UT, CH, CT, AS, MG, MN (38.1)	TG (16.7)	(0.0)
Middle	JK, KR, PY, AR, MZ (23.8)	MP, UP, PB (50.0)	TN, GJ, DL (75.0)
High	WB, KL, HR, OR, HP, AN, TR, GA (38.1)	RJ, AP (33.3)	MH (25.0)
**Literacy**			
Low	JK, BR, JH, CT, AS, ML, AR (33.3)	RJ, MP, UP, AP, TG (75.0)	(0.0)
Middle	WB, KA, HR, OR, UT, MN (28.6)	PB(25.0)	MH, GJ, TN (75.0)
High	KL, HP, CH, GA, AN,TR, PY, MZ (38.1)	(0.0)	DL(25.0)
**Scheduled Caste/Scheduled Tribe population**			
Low	OR, CT, HP, CH, MG, AR, MZ (33.3)	PB (16.7)	TN (25.0)
Middle	HR, AN, TR, PY, GA, MN (28.6)	RJ, MP, AP, TG (66.7)	GJ (25.0)
High	WB, JK, KR, BR, KL, JH, UT, AS (38.1)	UP (16.7)	TN, MH (50.0)
**Muslim population**			
Low	JK, BR, KL, HR, CH, AS, AN, PY, GA (42.9)	(0.0)	DL, TN (50.0)
Middle	WB,KR, UT, HP, MN (23.8)	RJ, UP, AP, PB, TG (83.3)	MH, GJ (50.0)
High	OR, JH, CT, TR, MG, AR, MZ (33.3)	MP (16.7)	(0.0)
**Total observations**	**21**	**6**	**4**

Note: State and union territories of Nagaland, Sikkim, Lakshadweep, Dadar & Nagar Haveli, and Daman & Diu had zero COVID-19 positive case; Figures in parentheses indicate the percent distribution of states and union territories by selected characteristics across low-, moderate-, and high-burden COVID-19 states. Appendix 1 provides details of abbreviated names of the state and union territories used above.

**TABLE 3:** Sub-national Analysis of COVID-19-Related Socioeconomic and Disease Exposure Patterns in India

Characteristics	Low-Burden COVID-19 Cluster	Moderate-Burden COVID-19 Cluster	High-Burden COVID-19 Cluster
**Population density**			
Low	JK, UT, CH, HP, AN, ML, MN, AR, MZ (42.9)	RJ (16.7)	(0.0)
Middle	KR, OD, JH, AS, TR, GA (28.6)	MP, AP, PB, TG (66.6)	MH, GJ(50.0)
High	WB, BR, KL, HR, CH, PY (28.5)	UP (16.7)	DL, TN(50.0)
**Slum population**			
Low	BR, KL, JH, AS, HP, ML, GA, MN, AR(42.9)	(0.0)	(0.0)
Middle	JK, KR, OR, UT, AN, TR (28.6)	RJ, UP, PB (50.0)	GJ (25.0)
High	WB, HR, CH, CT, PY, MZ (28.5)	MP, AP, TG (50.0))	MH, DL, TN (75.0)
**Urban population**			
Low	JK, BR, HR, OR, CT, AS, TR, MN, AR (42.9)	RJ, UP (33.3)	(0.0)
Middle	WB, JH, UT, HP, AN, MZ (28.6)	AP, PB, TG (50.0)	MH, GJ (50.0)
High	KR, KL, CH, MG, PY, GA (28.5)	MP (16.7)	DL, TN (50.0)
**Joint families**			
Low	CH,AS,AN,TR,MG,PY,GA,AR (38.1)	TG(16.7)	TN (25.0)
Middle	WB,JK,OR,CT,UT,MN,MZ (33.3)	UP, AP(33.3)	DL (25.0)
High	KR, BR, KL, HR, JH, HP(28.6)	RJ, MP, PB (50.0)	MH, GJ (50.0)
**Average person living per room**			
Low	JK, KL, UT AS, HP, AN, ML, GA, MZ (43.0)	(0.0)	(0.0)
Middle	WB, KR, HR, CH, CT, TR, PY, MN (38.0)	AP, PB, TG (50.0)	TN (25.0)
High	BR, OR, JH, AR (19.0)	RJ, MP, UP, (50.0)	MH, GJ, DL (75.0)
**Hand washing**			
Low	WB, BR, OR, JH, AS, TR (28.6)	RJ, UP, AP (50.0)	TN (25.0)
Middle	JK, KR, UT, CT, MG, MN, MZ (33.3)	MP, TG (33.3)	GJ (25.0)
High	KL, HR, CH, AN, GA, PY, AR (38.1)	PB (16.7)	MH, DL (50.0)

Note: Figures in parentheses indicate the percent distribution of states and union territories by selected characteristics across low, moderate, and high burden COVID-19 states. Appendix 1 provides details of abbreviated names of the state and union territories used above.

**TABLE 4:** Sub-national Analysis of COVID-19-Related Public Health Resilience Patterns in India

Characteristics	Low-Burden COVID-19 Cluster	Moderate-Burden COVID-19 Cluster	High-Burden COVID-19 Cluster
**Health index**			
Low	WB, KR, BR, JH, CT (23.8)	MP, UP, AP, TG (66.7)	MH, GJ (50.0)
Moderate	JK, KL, HR, OR, UT, AS, MG, MN (38.1)	RJ, PB (33.3)	TN (25.0)
High	CH, HP, AN, TR, PY, GA, AR, MZ (38.1)	(0.0)	DL(25.0)
**Mean per capita health expenditure**			
Low	WB,KR,BR,OR,JH(28.6)	MP,AP,UP,PB(67.0)	MH,GJ(50.0)
Moderate	KL,UT,CT,AS,TR,MG,MN(33.3)	RJ,TG(33.0)	DL,TN(50.0)
High	JK,CH,HP,AN,PY,GA,AR,MZ(38.0)	(0.0)	(0.0)
**Media exposure**			
Low	WB, JH,OR,BR, AS, TR, AR (33.3)	RJ, UP, MP(50.0)	(0.0)
Moderate	JK,HR,UT,CH,AN,MG(28.6)	AP, TG (33.3)	MH, GJ (50.0)
High	KR,KL,CH,HP,PY,GA,MN,MZ (38.1)	PB(16.7)	DL, TN (50.0)

Note: Figures in parenthees indicate the percent distribution of states and union territories by selected characteristics across low-, moderate-, and high-burden COVID-19 states. Appendix 1 provides details of abbreviated names of the state and union territories used above.

The relationship between education and COVID-19 infection indicates that states with a higher burden of COVID-19 infections have a moderate and higher literacy rate. Among demographic factors, age and co-morbidity are the pre-existing vulnerabilities that increase susceptibility to the disease. Among states with a moderate level of COVID-19 positive cases, a moderate and high percent of elderly population was observed. Half of the states with relatively high burden of COVID-19 positive cases also reported higher percent of the ageing population. Furthermore, with regard to the prevalence of morbidity, majority of the states reported a moderate burden of co-morbidities.

Sub-national analysis of socioeconomic and disease exposure patterns related to COVID-19 pandemic shows that states with a relatively higher burden of the disease reported moderate and higher population density (Table 3). Around three-fourths of the states reporting a higher number of infected cases such as Maharashtra, Delhi, and Tamil Nadu also had a relatively higher slum population. States with a moderate burden of infection had a moderate and higher concentration of slum population. The association of level of urbanization with the burden of COVID-19 shows that high disease burden states recorded a moderate and higher level of urbanization. With regard to the demographic structure of the family, it was found that half the high and moderate level infected states recorded a higher proportion of multi-generational families, whereas only 28% of low COVID-19 states recorded a higher number of a multi-generational families.

The sub-national analysis of public health resilience patterns related to COVID-19 pandemic shows that among the low COVID-19 burden states, approximately 24% recorded low scores in the health infrastructure index, while, 38% scored high (Table 4). Average health-care expenditure in low disease burden states was substantially higher than in states with a higher burden of disease. Almost 38% of low COVID-19 burden states spent more on public health, whereas, none of the moderate and high COVID-19 burden states came into this category.

### Multivariate Results

The descriptive analysis demonstrates substantial heterogeneities in the socioeconomic vulnerabilities associated with the burden of COVID-19 across states and union territories in India. We have fitted a negative binomial regression model to predict the risk of COVID-19 adjusting for demographic, socioeconomic, and health infrastructural characteristics across states and union territories in India (Table 5). The estimated regression model indicates that the proportion of elderly, the prevalence of any ailment, interstate migration, international migration, drinking of water outside household premises, health infrastructure, population density, proportion of urban population, proportion of nonagricultural casual laborers, and joint/extended families had a statistically significant and positive association with the risk of COVID-19 across states and union territories in India. For example, for 1-unit increase in the proportion of elderly population, and prevalence of any ailment were associated with 0.701-unit and 0.005-unit increase, respectively, in the COVID-19 risk, after adjusting for other socioeconomic, demographic, and health infrastructure characteristics. Likewise, the proportion of literates, hand washing before the meal and mass media exposure also had a statistically significant and negative association with COVID-19 risk. For instance, a 1-unit increase in literacy rate, hand washing before the meal and mass media exposure were associated with 0.306-, 0. 041-, and 1.236-unit decreases, respectively, in the COVID-19 risk across states and union territories in India.

**TABLE 5:** Estimated Negative Binomial Regression Coefficients Predicting the COVID-19 Risk by Selected Susceptibility, Exposure, and Resilient Characteristics Across the States/Union Territories in India

Characteristics	Coefficient	Standard Error	z-Value
Aging	0.701***	0.250	3.35
Any ailment	0.005***	0.001	0.14
Interstate migration	0.000***	0.000	4.80
International migration	0.000**	0.000	2.34
Literate	−0.306***	0.047	−6.53
Scheduled Caste/Scheduled Tribe population	−0.019	0.012	−1.58
Muslim population	−0.027	0.016	−1.68
Casual labor in agriculture	−0.103	0.104	−0.98
Casual labor in non-agriculture	0.108*	0.059	1.85
Poverty	0.000	0.030	0.02
Joint/extended family	0.090**	0.041	2.20
Average no. of person living per room	−1.069	0.762	−1.40
Household use for multi-purpose	0.174***	0.063	2.77
Handwash before meal	−0.041*	0.022	−1.84
Handwash before defecation	−0.006	0.017	−0.36
Drinking water outside premises	−0.047***	0.010	−4.53
Population density	0.000**	0.000	1.90
Proportion of urban population	0.034***	0.013	2.71
Proportion of slum population	0.346	0.107	3.23
Health infrastructure index	0.622***	0.186	3.35
Health expenditure	0.000	0.000	0.14
Mass media exposure	−1.236***	0.505	−2.45
Lnalpha	−0.755	0.275	
alpha	.469	.129	
N	36		
LR chi2(21)	90.22***		
Log-likelihood	−201.5395		
Likelihood-ratio test of alpha=0: chibar2(01) = 1359.91 Prob>=chibar2 = 0.000			

***P < 0.001.

**P < 0.05.

*P < 0.10.

### Assessment of Sub-national Level Vulnerabilities

**FIGURE 5:**
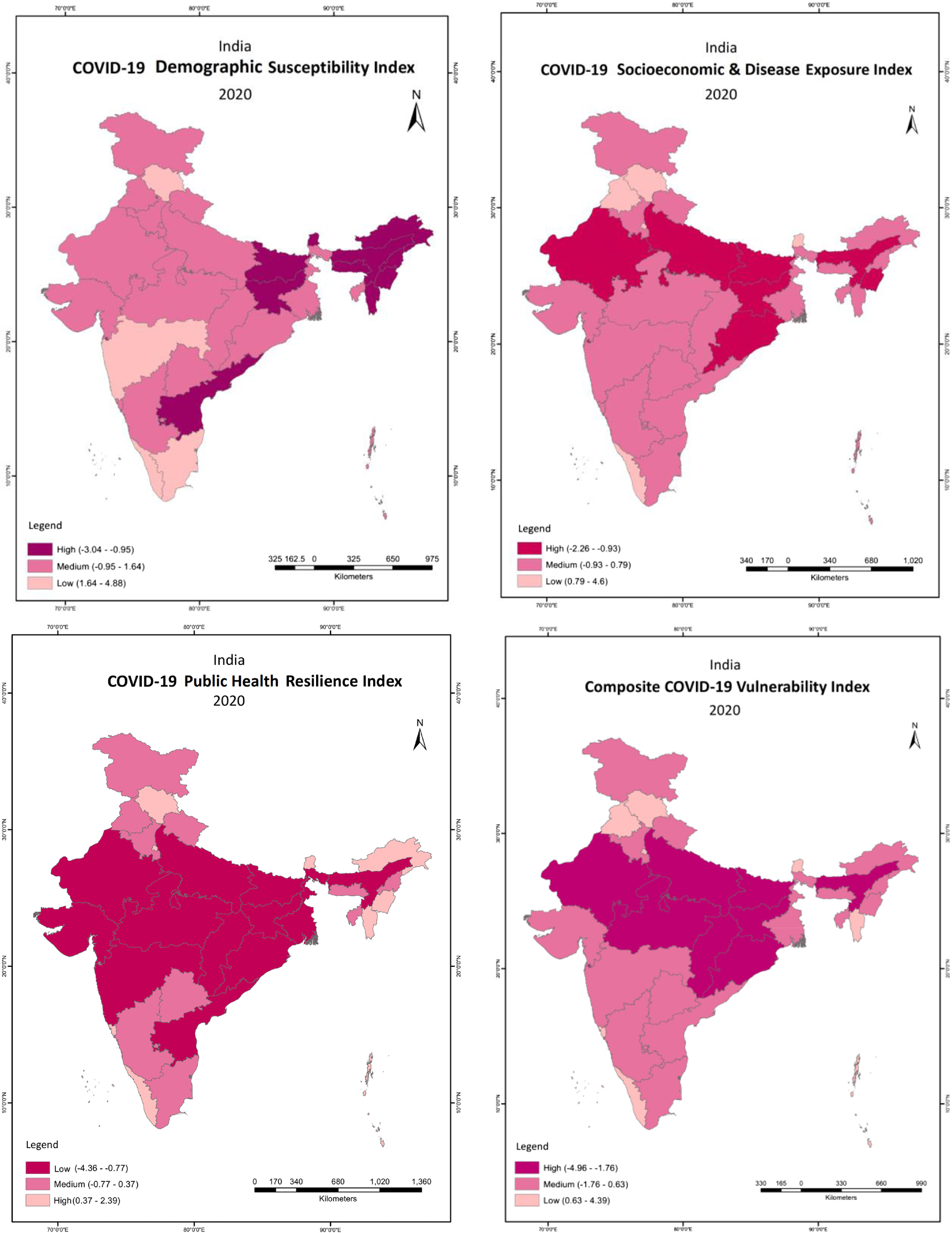
Estimated Indices of Susceptibility, Exposure, Resilience and Composite Vulnerability Index Related to COVID-19 Risk Across States and Union Territories in India

The composite vulnerability index at the sub-national level considering demographic susceptibility, socioeconomic and disease exposure, and public health resilience indicators suggest that the eastern states and parts of the northern states (accounting for 62% of India’s total population) were highly vulnerable to COVID-19 risk, and faced disproportionate challenges related to mitigation of this pandemic (Figure 5).

## DISCUSSION AND CONCLUSIONS

The present study makes a novel attempt to examine sub-national heterogeneities in the underlying socioeconomic vulnerabilities related to COVID-19 risk across states and union territories in India. Drawing upon the infectious disease vulnerability framework, the study attempts to quantify the COVID-19 risk and examined the potential policy solutions to mitigate the menace of this pandemic across states and union territories in India. Major findings of the study suggest that there persist substantial heterogeneities in the burden of COVID-19 positive cases, recovery rates, case-fatality rates and testing performance across Indian states. Overall, the COVID-19 risk has been increasing at an exponential rate, particularly in the high COVID-19 burden states of Maharashtra, Delhi, and Gujarat (an almost 90-fold increase during the study period). On the other hand, the medium COVID-19 burden states observed close to a 28-fold increase in the disease burden during the same period, followed by a 12-fold increase among the low COVID-19 burden states across India.

The unequal progression of COVID-19 across different states is an outcome of the complex interplay of the underlying demographic, socioeconomic, and health infrastructure heterogeneities. Examining the patterns of COVID-19 risk by the selected susceptibility factors across Indian states through the descriptive analysis, it was found that the high-burden COVID-19 states were characterized by relatively higher inter-state migration, international migration, a large proportion of elderly population, and an increased share of Scheduled Castes/Scheduled Tribes. In addition, analysis of the patterns of COVID-19 risk by the selected exposure factors across states suggest that the high-burden COVID-19 states had relatively higher population density, a disproportionately high share of urban and slum population, and a relatively higher average number of persons living per room.

Furthermore, the patterns of COVID-19 risk by the selected resilience factors highlights that the high-burden COVID-19 states were marked by relatively poor health infrastructure, lower public health expenditure, and high mass media exposure. This finding highlights that spatial heterogeneities associated with the resilience vulnerabilities might determine the course of mitigation and recovery prospects in the fight against COVID-19 risk across states in India. The composite index of vulnerabilities to COVID-19 risk indicates that the northern, central, and eastern states suffered from multidimensional vulnerabilities, and also exhibited limited resilience capabilities to tackle the COVID-19 risk. However, vulnerable population groups/states are dynamic, and those falling in these clubs may change over time owing to socially inclusive policy actions.^6^

The multivariate regression analysis confirmed that a disproportionately large share of elderly population, prevalence of co-morbidities, predominant inter-state and international migration, proportion of nonagricultural casual laborers, joint/extended families, multipurpose/business-related use of households, and level of urbanization had a statistically significant and positive association with COVID-19 risk across states and union territories in India. On the other hand, educational status, hand washing before meals, and exposure to mass media had a statistically significant and negative association with the COVID-19 risk across states and union territories in India. These findings have important implication for the mitigation of COVID-19 risk in India. The regression results revealed that older adults were more vulnerable to COVID-19 as compared with the rest of the population. Our finding is corroborated by various studies, which suggest that not only is the probability of the elderly getting infected with COVID-19 higher, but also the case fatality rate is found to be higher among them.^51-54^ It is obvious that states and union territories having a higher proportion of older adults have a higher burden of COVID-19 risk in comparison with other states. Therefore, there is need for policy action to encourage physical distancing among the elderly population. Apart from these social distancing policy measures, these states are also expected to divert more of its resources toward the care of older adults.

Our findings showed that the risk of COVID-19 was very high for those who were already suffering from pre-existing diseases, such as diabetes, heart disease, or respiratory problems. Our finding is in line with studies, which have suggested that co-morbidity is an important risk factor for COVID-19.^55-57^ These results are critical for those states and union territories with a higher proportion of older adults because co-morbidity is often found to be significantly higher among them. Therefore, it is imperative that the public health system is such that states are prepared to attend non-COVID related ailments with equal sensitivity.

The level of urbanization, population density, and slum population emerged as important risk factors for COVID-19 across states and union territories in India. Most of the highly urbanized, densely populated, and large slum cluster states and union territories have emerged as the hotspot of COVID-19 risk in India, including Maharashtra, Gujarat, Delhi and Tamil Nadu. This scenario is further aggravated in states with a higher volume of labor migration working in the informal sector. The large-scale inter-state migration mainly for informal casual work as observed from the analysis has emerged as an important risk factor of COVID-19 transmission across states. The migrants, who were invariably engaged in low profile jobs owing to their limited education, were less aware of the seriousness of the disease, and lived under suboptimal conditions, and hence, were at higher risk of being infected with COVID-19. Crowded living environments and congested living place limit the effective implementation of preventive measures, such as social distancing, and exposure to the disease was disproportionately higher for urban poor and labor migrants living in slums.^7,8,58^ Therefore, it is crucial to generate public awareness about the risk of COVID-19 and precautions to avoid the same. This is further supported by the findings related to safe sanitation practices and exposure to mass media that seem to be associated with significantly lower risk of COVID-19 in India. Efforts must be made to promote the use of safe sanitation practices and dissemination of accurate and verified information through television, radio, and newspaper related to restricting the spread of COVID-19.

Health infrastructure had a statistically significant and positive association with the COVID-19 risk across states and union territories in India. States with relatively robust health-care systems are better able to perform a large number of testing, treatments and recording of COVID-19 cases. In states with poor health infrastructure and limited testing facilities, the risk of COVID-19 tends to remain under-reported. This underscores the significance of a strong public health-care system to effectively implement preventive and curative measures and lower the risk of COVID-19 spread. It is essential to revitalize the public health system with the deployment of medical professionals, supply of personal protective equipment (PPE) kits, and intensive care unit (ICU) and ventilator facilities to meet the demand of COVID-19 patients as required. Public health system preparedness can go a long way toward mitigating the burden of COVID-19 risk in India.^59^

## APPENDIX 1 Classification of States and Union Territories According to the COVID-19 Confirmed Positive Cases in India

**TABLE 1:**

Low-Burden COVID-19	Medium-Burden COVID-19	High-Burden COVID-19
West Bengal (WB)	Madhya Pradesh (MP)	Maharashtra (MH)
Jammu & Kashmir (JK)	Rajasthan (RJ)	Gujarat (GJ)
Karnataka (KR)	Tamil Nadu (TN)	Delhi (DL)
Kerala (KL)	Uttar Pradesh (UP)	
Punjab (PJ)	Andhra Pradesh (AP)	
Bihar (BH)	Telangana (TG)	
Haryana (HR)		
Odisha (OR)		
Jharkhand (JH)		
Chandigarh (CD)		
Uttarakhand (UT)		
Assam (AS)		
Chhattisgarh (CT)		
Himachal Pradesh (HP)		
Andaman and Nicobar Islands (ANI)		
Puducherry (PY)		
Goa (GA)		
Manipur (MN)		
Tripura (TR)		
Arunachal Pradesh (AR)		
Mizoram (MZ)		
